# Penile Fracture: Simultaneous Complete Urethral Rupture with Bilateral Corpora Cavernosa Rupture

**DOI:** 10.1155/2018/4929518

**Published:** 2018-09-25

**Authors:** Ibrahim Alnadhari, Osama Abdelhaleem Abdeljaleel, Venkata Ramana Pai Sampige, Ausama Abdulmuhsin, Ahmad Shamsodini

**Affiliations:** Division of Urology, Surgery Department, Al Wakra Hospital, Hamad Medical Corporation, Doha, Qatar

## Abstract

Penile fracture is not uncommon blunt trauma to the penis. Here, we present a rare case of penile fracture during sexual intercourse. The patient presented with penile swelling, bleeding per urethra, and inability to pass urine. Retrograde urethrogram showed significant extravasation of contrast from anterior penile urethra and no contrast passing to proximal urethra. Surgical exploration showed complete urethral rupture and bilateral cavernosal rupture. This case represents the value of urethrogram to evaluate the urethral injury and the association of complete urethral injury with bilateral ventral cavernosal injury.

## 1. Introduction

Penile fracture is caused by blunt injury to erected penis leading to rupture of the tunica albuginea of the corpus cavernosum. Mobility of the penis and its protected location make such injury rare. The most common cause of penile fracture is hitting erected penis against the perineum or the symphysis pubis during vigorous intercourse [1, 2]. Penile fracture can be associated with urethral injury in 6-9% [1, 2]. We present a rare case of complete urethral rupture with rupture of both corpora cavernosa.

## 2. Case Summary

A 62-year-old male patient with a known case of diabetes mellitus presented to the emergency with history of penile swelling for 4 hours. The incident happened during sexual intercourse in doggy position, while he was hurrying to reinsert his penis into the vagina, he struck the perineum of his partner. He suddenly heard a cracking sound and severe pain in the penis followed by swelling of the penis, sudden detumescence, and blood coming from urethra associated with inability to pass urine.

Physical examination showed severely swollen, deformed penis with deviation of the distal shaft of penis toward the dorsal surface and blood coming from the urethral meatus. Palpable hematoma with tenderness was noted over the ventral aspect of distal shaft of the penis.

**Figure 1:**
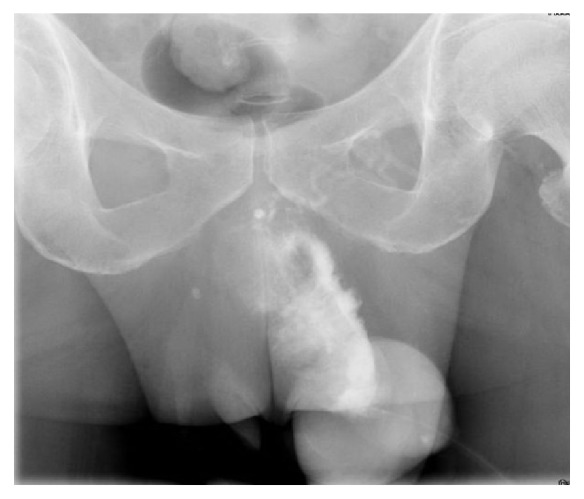
Retrograde urethrogram showed significant extravasation of contrast from anterior penile urethra to surrounding soft tissue.

Retrograde urethrogram showed significant extravasation of contrast from anterior penile urethra to surrounding soft tissue and no contrast passing to proximal urethra, likely penile urethra injury (Figure 1).

**Figure 2:**
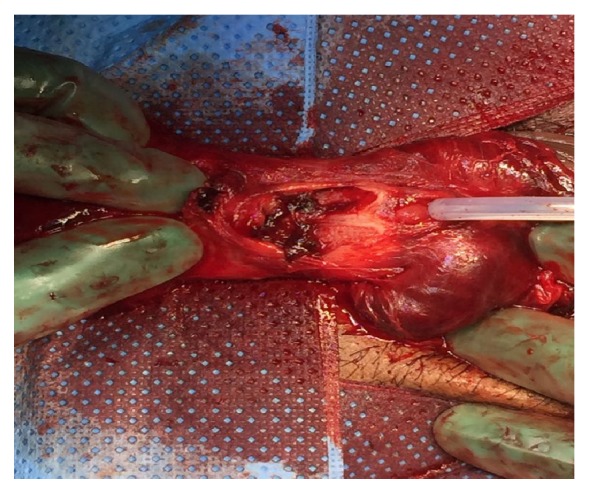
Intraoperative finding of bilateral corpora cavernosa with complete urethral rupture.

Surgical exploration was done through subcoronal circumferential incision. The penis was degloved, and evacuation of hematoma was carried out. There were two large defects on the ventral aspect of both corpora cavernosa with complete rupture of the urethra at the mid penile shaft (Figure 2).

**Figure 3:**
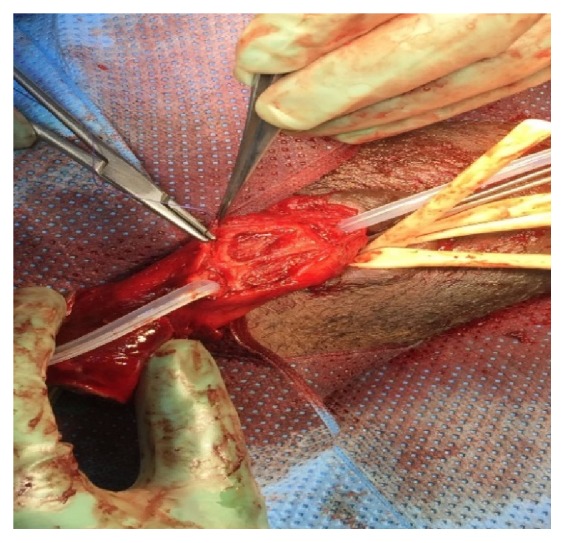
Closure of corpora cavernosa tears.

**Figure 4:**
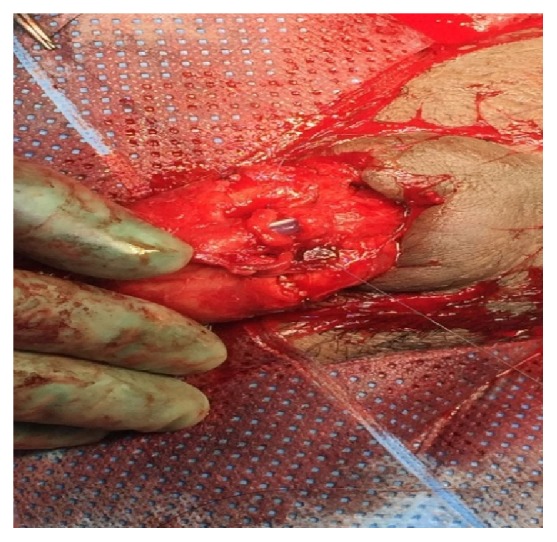
End to end urethral anastomosis over urethral catheter.

A 16 F urethral catheter was inserted in the proximal urethra followed by tourniquet application on the base of penis and mobilization of both urethral ends. Closure of the corpora cavernosa on both sides was done by use of 3/0 vicryl suture (Figure 3). Subsequently, end to end, mucosa to mucosa, and tension-free urethral anastomosis over the 16 F urethral catheter was done using 4/0 vicryl suture (Figure 4). Some reinforcement suture in the corpus spongiosum was done.

**Figure 5:**
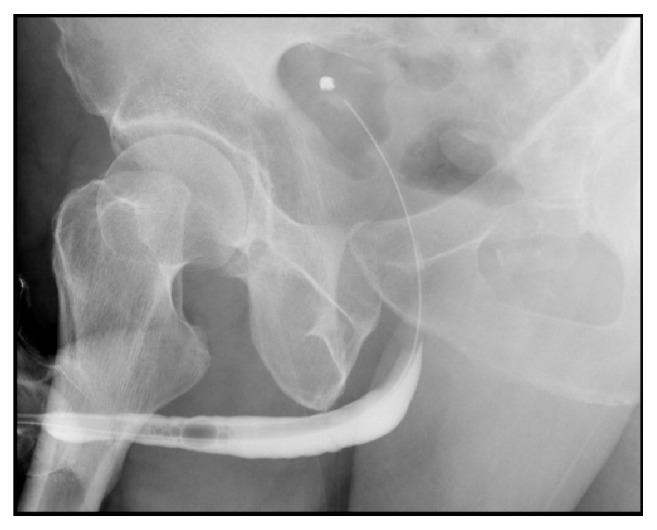
Pericatheter urethrogram showed urethral continuity with no extravasation.

Wound was closed in two layers for dartos and skin. As there was urethral involvement, patient was given cefuroxime 1.5 gm intravenous TID started on anesthesia induction for one day and discharged on cefuroxime 500 mg BID for one week. Patient was discharged next day and urethral catheter kept for 4 weeks after which a pericatheter urethrogram showed urethral continuity with no extravasation (Figure 5) and the patient passed urine freely after removal of the catheter.

Patient reported good erection with no curvature of the penis and good urine flow (Qmax 20 ml/sec with 20 ml post-voiding residual) during follow-up visits after 4 months. The patient was scheduled for further follow-up visits to evaluate the long term results.

## 3. Discussion

The thickness of tunica albuginea covering the corpus cavernosum depends on the state of the penis which reaches up to 2.4 mm in flaccid state, while in the erect state it can reaches 0.25-0.5 mm [3]. Thin tunica albuginea, in addition to sudden bending of the erected penis, resulted in raising intracavernosal pressure to around 1500 mm which leads to rupture of the tunica albuginea and penile fracture [3].

The causes of penile fracture have some geographic variation but the most common cause is hitting of the penis outside the vagina on the perineum or the pubis symphysis during sexual intercourse, and other causes include masturbation, rolling over erected penis, and forced flexion [1, 2]. Some studies showed that the most common cause is a habit by people in Middle East called taghaandan which is bending of the erected penis to achieve tumescence [4, 5].

Associated urethral injuries vary from 6% to 9 % [1, 2]. A reported complete urethral rupture is rare and mostly associated with bilateral corporeal rupture [6]. Bleeding per urethra is an indicator for urethral injuries but its absence dose not exclude urethral injuries [2]. Retrograde urethrogram is used to confirm urethral injury in most of the studies [1, 2, 4]. Flexible cystoscopy during surgical procedure was used as diagnostic tool for urethral injury [7].

Most of the patient give symptoms including cracking sound followed by sudden detumescence with painful penile swelling. This classic history with local finding of eggplant picture of the penis and tenderness at the site of rupture makes the clinical diagnosis of penile fracture without need for further diagnostic tests in most situations [1, 6]. Penile ultrasound, cavernosography, and MRI can be helpful in some cases [1, 2, 8].

Surgical intervention has been shown to have better outcome and shorter hospital stay with less complications rate of erectile dysfunction, penile curvature, and penile pain on erection in comparison to the conservative management [1, 2, 4]. Urethral injury management depends on the extent of the injury. Minimal urethral injuries can be managed with urinary diversion or direct suture of the tear, but in severe urethral injury or complete urethral rupture, spatulated, mucosa to mucosa, tension-free anastomosis over urethral catheter is needed [1, 9]. Additionally, urinary diversion via a suprapubic tube may be considered. Delayed repair with a graft may be considered, and delayed repair can be carried out several weeks later under controlled circumstances and after resolution of the hematoma.

## 4. Conclusion

Penile fracture is urological emergency, and clinical diagnosis is sufficient in most of the cases. Ascending urethrogram is useful in diagnosis of urethral injury, and the degree of urethral injuries depends on the severity of the injury. Complete urethral rupture is mostly associated with bilateral corporal rupture as in our case and immediate surgical exploration with repair being mandatory.

## References

[1] Ibrahiem E. H. I., El-Tholoth H. S., Mohsen T., Hekal I. A., El-Assmy A. (2010). Penile Fracture: Long-term Outcome of Immediate Surgical Intervention. *Urology*.

[2] Amer T., Wilson R., Chlosta P. (2016). Penile fracture: A meta-analysis. *Urologia Internationalis*.

[3] Bitsch M., Kromann-Andersen B., Schou J., Sjontoft E. (1990). The elasticity and the tensile strength of tunica albuginea of the corpora cavernosa. *The Journal of Urology*.

[4] Zargooshi J. (2000). Penile fracture in Kermanshah, Iran: report of 172 cases. *The Journal of Urology*.

[5] Al Ansari A., Talib R. A., Shamsodini A., Hayati A., Canguven O., Al Naimi A. (2013). Which is guilty in self-induced penile fractures: Marital status, culture or geographic region? A case series and literature review. *International Journal of Impotence Research*.

[6] Hoag N. A., Hennessey K., So A. (2011). Penile fracture with bilateral corporeal rupture and complete urethral disruption: case report and literature review. *Canadian Tax Journal*.

[7] Kamdar C., Mooppan U. M. M., Kim H., Gulmi F. A. (2008). Penile fracture: Preoperative evaluation and surgical technique for optimal patient outcome. *BJU International*.

[8] Pavan N., Tezzot G., Liguori G. (2014). Penile fracture: Retrospective analysis of our case history with long-term assessment of the erectile and sexological outcome. *Archivio Italiano di Urologia e Andrologia*.

[9] Tsang T., Demby A. M. (1992). Penile fracture with urethral injury. *The Journal of Urology*.

